# Abnormal neutrophil-to-lymphocyte ratio in children with autism spectrum disorder and history of maternal immune activation

**DOI:** 10.1038/s41598-023-49789-5

**Published:** 2023-12-16

**Authors:** Pierre Ellul, Anna Maruani, Hugo Peyre, Valérie Vantalon, Daphnée Hoareau, Hugo Tiercelin, Michelle Rosenzwajg, David Klatzmann, Richard Delorme

**Affiliations:** 1 Child and Adolescent Psychiatry Department, Robert Debre Hospital, APHP, 48 Boulevard Serurier, 75019 Paris, France; 2 UMRS_959, Immunology-Immunopathology-Immunotherapy (i3), Sorbonne Université/INSERM, Paris, France; 3 Human Genetics and Cognitive Functions, Institut Pasteur, Paris, France

**Keywords:** Immunology, Biomarkers, Psychiatric disorders, Autism spectrum disorders

## Abstract

Maternal immune activation (MIA), related to autoimmune/inflammatory diseases or acute infections, during the two first trimesters of pregnancy is a risk factor for autism spectrum disorders (ASD) in offspring. In mice, MIA has a long-term impact on offspring’s immune equilibrium resulting in a pro-inflammatory phenotype. We therefore hypothesized that children with ASD and a history of MIA could display a similar phenotype specifically assessed by a higher neutrophil to lymphocyte ratio (NLR). In this study, we used a retrospective sample of 231 dyads involving children with ASD and their mothers. Among ASD patients, 12% had a history of MIA. The multivariate analysis revealed a significant association between NLR in children with ASD and maternal history of MIA (F = 2.27, p = 0.03). Using a categorical approach, we observed an abnormal NLR (over 3) in 7.4% of children with ASD MIA+ compared to 1.9% for MIA−. Our study supports the hypothesis suggesting an impact of MIA on the risk of ASD. Further studies could contribute to the development of biomarkers in MIA+ ASD and enable the development of targeted immunomodulatory therapies.

## Introduction

Autism spectrum disorders (ASD) are a heterogeneous group of neurodevelopmental disorders^1^ with an estimated prevalence of approximately 1 in 100^2^. ASD are characterized by a deficit in social communication associated with restricted and repetitive behaviors^3^. ASD result of a complex interplay between genetics, epigenetics and environmental factors^4^.

Preclinical and epidemiological studies highlight the role of maternal immune activation (MIA) during pregnancy, whether due to autoimmune/inflammatory diseases or acute infections, as a risk factor for ASD in the offspring^5^. Mice models show the central role of maternal cytokines in the disruption of fetal brain development. The maternal secretion of interleukin 6 (IL6) and 17a (IL17a) during gestation plays a pivotal role in the development of ASD-like behaviors in pups^6,7^. MIA exposure also induces long-lasting changes in the offspring’s immune system. In utero exposure to MIA leads to increased activation of Th17 lymphocytes—a pro-inflammatory subset secreting IL17a—and a decrease of the regulatory T lymphocytes (Tregs), in the offspring through potential epigenetic mechanisms^8,9^. Similar dysregulation were recently reported in children of women who were affected by the SARS-Cov2 during pregnancy^10^.

The neutrophil/lymphocyte ratio (NLR) is an indicator of impaired cell-mediated immunity, frequently associated with inflammation^11^ and could represent an immune marker in the offspring in case of MIA during pregnancy. Higher NLR have been correlated to negative prognoses in several human diseases such as cardiovascular diseases, kidney diseases, infections or chronic respiratory disorders, and is also associated with all-cause mortality in the general population^12,13^. In recent years, there is a growing interest in NLR in psychiatric disorders suspected to be related to an immune dysregulation such as schizophrenia, bipolar disorders or depression^14,15^. To our knowledge, few studies examined NLR in ASD, but with inconclusive results^16–18^. None of these reports however assessed the impact of MIA in the NLR variability.

In the present study, we retrospectively explored the NLR in children with ASD and a history of active MIA during pregnancy. We hypothesized that offspring with ASD and MIA (MIA+) would display an increased NLR compared to those with ASD without MIA (MIA−).

## Methods

### Participants

We included in our study children with ASD who were part of the PARIS (Paris Autism Research International Sib-pair) study, conducted by the Excellence Centre for Autism & Neuro-developmental Disorders (InovAnd) at the Robert Debré Hospital between March 2017 to April 2021. This study was approved by the local ethics committee (2021-27 No. IDRCB: 2021-A00489-32). Informed consents were obtained from all participants and/or their legal guardians before enrollment in the study.

Pre- and peri- natal history was evaluated through a direct semi-structured interview with the mother of each child enrolled in the study. The final diagnosis of ASD was performed according to DSM-5 criteria^3^ by summing up the information from the Autism Diagnostic Interview-Revised^19^, the Autism Diagnostic Observation Schedule -2nd edition (ADOS-2)^20^ and clinical records of the individuals. We paid a specific attention to a history of MIA by scrutiny explore any diagnosed immune mediated illnesses which could have occurred during pregnancy. This interview was conducted by a child and adolescent psychiatrist specializing in ASD, at best in the presence of both parents, but often only the mother. We focused on any history of MIA during pregnancy based solely on the parents' declaration. Based on this information, children were then split either in MIA (MIA+) or in non-MIA (MIA−) sub-groups. We considered mothers with a significant history of a MIA-related event during pregnancy when they were: (i) with an autoimmune disease as listed by the American Autoimmune Related Diseases Association (https://autoimmune.org/disease-information/). The disease should have occurred during the first or the second trimester of pregnancy, or was present before the pregnancy and had a flare-up requesting a treatment adjustment during pregnancy; (ii) with a viral or bacterial infection during pregnancy with a fever over 38.5 °C for more than 24 h. Occurring during the first or the second trimester of pregnancy. Mothers with an infection resulting from a pathogen with a well-documented direct brain cytopathic effect (such as cytomegalovirus infection) were excluded^21^ (iii) with gestational diabetes. We only considered mothers requiring insulin supplementation. We considered that this condition was more likely to be associated with a significant systemic metabolic inflammation^18^.

All research was performed in accordance with relevant guidelines/regulations and in accordance with the Declaration of Helsinki.

### Neutrophil to lymphocyte ratio

Following clinical assessments, patients diagnosed with ASD were sampled by a nurse. The blood was then sent directly to the laboratory at room temperature for analysis. Routine blood counting was performed using XN 3000 (Sysmex) NLR was then calculated by dividing the absolute value of the neutrophil count by the absolute value of the lymphocyte count. In the categorical analysis, a NLR greater than 3 was considered as pathological^22^.

### Statistical analysis

Before each analysis, the normality of the distribution of the variables was tested with a Shapiro–Wilk test. Parametric or non-parametric tests were used accordingly. For continuous variables, we used independent sample t-tests, or Wilcoxon for continuous variables. For categorical variables, the Fisher test was used.

In linear models, only models with a normal distribution of residuals were used. Hierarchical regression analysis are in Supplementary Table 1. The final model of multivariate analysis was thus adjusted on age, gender and pregnancy complications (placenta previa and maternal–fetal infections). Statistical analysis was performed using R studio version 4.2.1.

## Results

### General characteristics of the offspring with ASD

Among the 231 mother–child dyads included in the study, a MIA during pregnancy was found in 11.68% of mothers (n = 27) (MIA+) (Supplementary Table 2).

Dyads without MIA during pregnancy (n = 204, 88.31%) were used as a comparison group (MIA−). Demographics characteristics of the population were provided in Table 1. The mean age of offspring with ASD was significantly lower in the MIA+ group (78.2 ± 24.8 vs 89.6 ± 25.9 months, *p* = 0.033). In accordance with literature, we observed that mothers from MIA+ group have had more pregnancy complications than those in the MIA- group, with significant differences for placenta previa (p = 0.03) and maternofetal infection at birth (p = 0.02)^23^. Concerning offspring, we found that offspring from MIA+ have a lower birth height (p = 0.04). We did not report any other significant difference concerning birth parameters and offspring’s medical history.

**Table 1:** Main characteristics of autism spectrum disorder patients with or without a history of maternal immune activation.

	MIA+	MIA−	*p* value
Numbers of patients	27	204	
Offspring			
Male/female, n (%)	22/5 (81/19)	159/44 (78/22)	0.89
Age at inclusion—month [mean (SD)]	78.22 (24.84)	89.55 (25.89)	0.03*
Pregnancy complications, n (%)			
Consanguinity	0 (0)	16 (7.8)	0.22
Medically-assisted procreation	0 (0)	12 (5.88)	0.36
History of spontaneous miscarriage	10 (37.03)	43 (21.07)	0.1
Folate supplementation	16 (59.25)	86 (42.15)	0.14
Threat of premature delivery	1 (3.70)	8 (3.92)	1
Arterial hypertension	1 (3.70)	9 (4.41)	1
Placenta previa	2 (7.40)	1 (0.49)	0.03*
Premature rupture of membranes	2 (7.40)	2 (0.98)	0.06
Intrauterine growth retardation	1 (3.70)	7 (3.43)	1
Macrosomia	0 (0)	2 (0.98)	1
Maternal-foetal infection	3 (11.1)	3 (1.47)	0.02*
Birth parameters, mean (SD)			
Birth term	38.53 (1.39)	38.58 (2.23)	0.06
Birth height	48.84 (2.34)	49.46 (3.54)	0.04*
Birth weight	3273 (452.01)	3289 (658.24)	0.47
Head circumference	35.62 (3.78)	34.76 (3.02)	0.31
1-min Apgar	9.44 (1.08)	9.44 (1.49)	0.43
5-min Apgar	9.88 (0.42)	9.87 (0.73)	0.51
Biological parameters (univariate analysis)			
Neutrophils	3.30 (1.89)	3.15 (1.35)	0.90
Lymphocytes	3.18 (1.70)	3.02 (1.12)	0.94
Neutrophils/lymphocytes	1.42 (1.63)	1.17 (0.73)	0,81

*MIA* maternal immune activation, *SD* standard deviation.

*p < 0.05.

### NLR variability

We first explored the NLR variability by performing a univariate analysis. We observed no significant difference between the MIA+ and MIA− sub-groups considering neutrophils (3.30 ± 1.89 × 10^9^/L vs 3.15 ± 1.35 × 10^9^/L; p = 0.68), lymphocytes (3.18 ± 1.7 × 10^9^/L vs 3.02 ± 1.12 × 10^9^/L; p = 0.63) or the NLR (1.42 ± 1.63 vs 1.17 ± 0.73; p = 0.43).

We then performed a multivariate analysis by incorporating potential confounding factors (Table 2).

**Table 2:** Details of multivariate linear regression analysis.

	Beta	t	p-value
Lymphocytes			
Model 1			
MIA	0.04	0.65	0.51
Model 2			
MIA	0.009	0.14	0.89
Age	−0.22	−3.32	0.001
Gender	0.06	0.89	0.38
Model 3			
MIA	0.005	0.08	0.94
Age	−0.21	−3.22	0.001
Gender	0.05	0.75	0.45
MFI	−0.06	−0.95	0.34
Placenta previa	0.09	1.29	0.2
Neutrophiles			
Model 1			
MIA	0.034	0.52	0.6
Model 2			
MIA	0.06	0.89	0.37
Age	0.16	2.47	0.01
Gender	0.004	0.06	0.95
Model 3			
MIA	0.08	1.15	0.25
Age	0.17	2.56	0.01
Gender	0.003	0.043	0.97
MFI	−0.05	−0.77	0.44
Placenta previa	−0.04	−0.63	0.53
NLR			
Model 1			
MIA	0.09	1.38	0.17
Model 2			
MIA	0.12	1.91	0.06
Age	0.21	3.19	0.002
Gender	−0.08	−1.31	0.19
Model 3			
MIA	0.14	2.14	0.03*
Age	0.21	3.21	0.001
Gender	−0.08	−1.26	0.21
MFI	−0.02	−0.33	0.74
Placenta previa	−0.08	−1.15	0.25

*MIA* maternal immune activation, *MFI* materno-fetal infection.

*p < 0.05.

We found a significant increase of the NLR in the MIA+ offspring (F = 3.04, p = 0.03) but not with the concentrations of lymphocytes (p = 0.94) and neutrophils (p = 0.25).

Using a categorical approach, there was a tendency for more frequent pathological value of NLR (NLR over 3) in the MIA+ than in the MIA− subgroups, although this did not reach significance. [7.4% (2/27) vs 1.9% (4/204), p = 0.14].

## Discussion

In accordance with our initial hypothesis, we reported that NLR was significantly higher in ASD children with a history of MIA than in ASD without MIA. Our results fostered preliminary evidence suggesting that ASD patients with a history of MIA have persistent peripheral inflammation and that NLR may be a potential biomarker of this immune dysregulation.

ASD by itself are associated with an immune dysregulation such as a decrease in Tregs and an increase in Th17^24,25^. In animal models of ASD, MIA also induced long term increase of the Th17^8^ and decrease of Tregs in offspring^9^. This Tregs/Th17 imbalance results in a polarization of the immune balance toward a peripheral inflammation. Although we lack a control group of children without ASD born to mothers with MIA to independently assess MIA impact on the NLR of the offspring, recent basic neuroimmunology studies emphasized the importance of IL-17a in brain development but also in the homeostasis of cognitive function^26,27^. Also, Tregs have been recently discovered in the perineuronal net and could play a fundamental role in neuronal homeostasis^28^. Moreover, in a mouse model of MIA-induced ASD, Tregs transplantation leads to normalization of autistic behaviors^29^. These data highlight that the immune dysregulation induced by MIA could have a pathophysiological impact in ASD children. This modification of the Tregs/Th17 balance potentially leads to a chronic pro-inflammatory state in ASD patients exposed to MIA, which should be measurable in peripheral blood.

Using NLR, our study was the first to confirm preclinical findings in autistic individuals with a history of MIA. To our knowledge, only two previous studies have explored NLR in ASD. They reported no association with ASD but without considering the heterogeneity of individuals regarding MIA^16,17^. The authors however showed a trend for a significant correlation between NLR and autistic symptom severity. Interestingly, MIA during pregnancy was also associated with severe ASD related features in the offspring^30^. Taken together with the literature, our results highlighted the potential role of immunity dysregulation in the pathophysiology of ASD.

In our sample, we also observed more pregnancy complications in the MIA+ group. Numerous examples in the literature emphasize that neurodevelopmental disorders may emerge through the addition of causal factors^5^. In animal studies, MIA is considered as a disease primer, making the offspring more susceptible to a second hit that might precipitate the rise of neurodevelopmental disorders (prematurity, early post-natal infection)^31^. The overrepresentation of perinatal hitches in the MIA+ individuals may indirectly reflect this additive model of determinism in ASD^29^. Although no studies have investigated this hypothesis in the context of MIA, future cohort studies may try to further decipher the intrinsic link between MIA in pregnant mothers and the development of ASD in offspring. We advocate that they adopt a longitudinal perspective and take into account (i) 'secondary' events, (ii) clinical phenotyping of patients (severity of autism co-morbidity, ADHD co-morbidity) and (iii) include a deep immunophenotyping approach on larger samples^32^. Our study has to be considered in light of its limitations. First, due to the intrinsic nature of our study, the collection of data and in particular events during pregnancy was retrospective and may therefore be subject to recall bias. Secondly, our sample size was small, particularly for the MIA+ group. These two limitations were balanced by the use of stringent criteria on the definition of active MIA during pregnancy allowing (i) to temper recall bias and (ii) to define a homogeneous and powerful MIA+ sample, enough to detect subtle differences between groups., in the general population infection during pregnancy is very frequent^33^. Thus, trying to make association between this common events with a less common occurrence (ASD in the offspring) carries the risk of false associations. It is important to note, however, that our results are consistent with those found in basic research studies. Lastly, the NLR cut-off used in categorical analysis is only validated in an adult population. To our knowledge, no such threshold exists for the pediatric population^34^. However, with regard to a dimensional approach, several studies have shown its value in the paediatric population, whether in neonatology^35^, or for cardiovascular^36^, infectious^37^ or neurological pathologies^38^.

## Conclusions

NLR is a validated pediatric tool for assessing peripheral inflammation. In ASD, using NRL, we showed that history of MIA seems to be associated with long term peripheral immune dysregulation. Further studies are needed to define if and how this peripheral inflammation contributes to the pathophysiology of autism. Indeed, there is still a lack of precise data on the interaction between peripheral inflammation and brain function in ASD. However, we can hope that the translation of knowledge gained from animal models to patients, using for example deep immunophenotyping data, will allow to fill gaps in our knowledge and open new avenues in the development of immunotherapy in ASD.

### Supplementary Information

Supplementary Tables.

## Data Availability

The datasets and the code used during the current study are available from the corresponding author on reasonable request.
