# Supplementary material for: Abnormal neutrophil-to-lymphocyte ratio in children with autism spectrum disorder and history of maternal immune activation
Source: Sci Rep. 2023 Dec 16;13:22424. doi: 10.1038/s41598-023-49789-5 (PMC10725503; doi:10.1038/s41598-023-49789-5)
Supplement: Supplementary file 1 — Supplementary Tables. [file 41598_2023_49789_MOESM1_ESM.docx]

**Supplementary Table 1.** Hierarchical regression analysis of MIA

| **Lymphocyte and MIA : Independant variable** | **R2** | **Beta** | **t** | **p-value** |
| --- | --- | --- | --- | --- |
| **Model 1** | 0,05 |  |  |  |
| MIA |  | 0,01 | 0,16 | 1,86 |
| Age |  | -0,21 | -3,3 | 0,0012 |
| **Model 2** | 0,004 |  |  |  |
| MIA |  | 0,04 | 0,64 | 0,52 |
| Gender |  | 0,05 | 0,71 | 0,48 |
| **Model 3** | 0,007 |  |  |  |
| MIA |  | 0,06 | 0,86 | 0,39 |
| MFI |  | -0,08 | -1,11 | 0,27 |
| **Model 4** | 0,007 |  |  |  |
| MIA |  | 0,03 | 0,43 | 0,66 |
| Placenta previa |  | 0,07 | 1,06 | 0,29 |
|  |  |  |  |  |
| **Neutrophils and MIA : Independant variable** | **R2** | **Beta** | **t** | **p-value** |
| **Model 1** | 0,03 |  |  |  |
| MIA |  | 0,06 | 0,9 | 0,37 |
| Age |  | 0,16 | 2,48 | 0,01 |
| **Model 2** | 0,001 |  |  |  |
| MIA |  | 0,03 | 0,52 | 0,6 |
| Gender |  | 0,01 | 0,18 | 0,86 |
| **Model 3** | 0,003 |  |  |  |
| MIA |  | 0,04 | 0,63 | 0,53 |
| MFI |  | -0,04 | -0,61 | 0,54 |
| **Model 4** | 0,003 |  |  |  |
| MIA |  | 0,04 | 0,65 | 0,52 |
| Placenta previa |  | -0,05 | -0,71 | 0,48 |
|  |  |  |  |  |
|  |  |  |  |  |
| **NLR and MIA : Independant variable** | **R2** | **Beta** | **t** | **p-value** |
| **Model 1** | 0,05 |  |  |  |
| MIA |  | 0,12 | 1,87 | 0,06 |
| Age |  | 0,2 | 3,12 | 0,002 |
| **Model 2** | 0,01 |  |  |  |
| MIA |  | 0,09 | 1,41 | 0,16 |
| Gender |  | -0,07 | -1,13 | 0,26 |
| **Model 3** | 0,008 |  |  |  |
| MIA |  | 0,09 | 1,38 | 0,17 |
| MFI |  | -0,008 | -0,12 | 0,9 |
| **Model 4** | 0,01 |  |  |  |
| MIA |  | 0,11 | 1,6 | 0,11 |
| Placenta previa |  | -0,08 | -1,2 | 0,23 |

**Supplementary Table 2**: Frequencies of maternal immune events in the MIA+ group (n = 27 of 231 participants).

| **Immune Condition** | **n (% of total cohort)** |
| --- | --- |
| **Autoimmune conditions** |  |
| Gestational diabetes | 8 (29.6) |
| Hashimoto’s thyroiditis | 5 (18.5) |
| Systemic Lupus Erythematosus | 3 (11.1) |
| Type 1 diabetes | 2 (7.4) |
| Crohn’s disease | 1 (3.7) |
| Antiphospholipid syndrome | 1 (3.7) |
| **Infection** |  |
| Pyelonephretis | 1(3.7) |
| AIDS | 1 (3.7) |
| Lung infection | 1 (3.7) |
| Flu | 1 (3.7) |
| Scabies | 1 (3.7) |
